# Supplementary material for: Protein Quality Control Activation and Microtubule Remodeling in Hypertrophic Cardiomyopathy
Source: Cells. 2019 Jul 18;8(7):741. doi: 10.3390/cells8070741 (PMC6678711; doi:10.3390/cells8070741)
Supplement: Supplementary file 1 [file cells-08-00741-s001.pdf]

Article

Protein quality control activation and microtubule remodeling in hypertrophic cardiomyopathy

Larissa M. Dorsch[1]*, Maike Schuldt[1], Cristobal G. dos Remedios[2], Arend F.L. Schinkel[3], Peter L. de Jong[4], Michelle Michels[3], Diederik W.D. Kuster[1], Bianca J.J.M. Brundel[1] and Jolanda van der Velden[1,5]

1 Amsterdam UMC, Vrije Universiteit Amsterdam, Department of Physiology, Amsterdam Cardiovascular Sciences, Amsterdam, the Netherlands; l.dorsch@vumc.nl, m.schuldt@vumc.nl, d.kuster@vumc.nl, b.brundel@amsterdamumc.nl, j.vandervelden@vumc.nl
2 Sydney Heart Bank, Discipline of Anatomy, Bosch Institute, University of Sydney, Sydney, Australia; crisdos@anatomy.usyd.edu.au
3 Department of Cardiology, Thoraxcenter, Erasmus Medical Center, Rotterdam, the Netherlands; a.schinkel@erasmusmc.nl, m.michels@erasmusmc.nl
4 Department of Cardiothoracic Surgery, Thoraxcenter, Erasmus Medical Center, Rotterdam, the Netherlands; p.l.dejong@erasmusmc.nl
5 Netherlands Heart Institute, Utrecht, the Netherlands
* Correspondence: l.dorsch@vumc.nl; Tel.: +31-204448110

Received: 21.05.2019; Accepted: date; Published: date

Supplementary Materials: The following are available online at www.mdpi.com/xxx/s1,

Multivariate generalized linear model

Figure S1: Controls.

Figure S2: Uncropped full-width pictures of Western blotting membranes.

Figure S3**.** Correlation of HSPs levels and α-tubulin.

Materials and Methods

Multivariate generalized linear model

Multivariate generalized linear model was performed with SPSS Statistics version 22.0 by requesting from the SPSS menu selections Analyze > General Linear Model > Multivariate. In the initial multivariate GLM dialog, the key PQC players were selected as Dependent Variables, mutation_group and sex as Fixed Factors and age_at_operation as Covariate. In the Model button dialog the model was specified as Custom to study Main effects. All Factors & Covariates (mutation_group, sex, age_at_operation) were included in the model, Type III Sum of squares was determined and Include intercept in model was ticked. In the Contrasts button dialog the Contrast was changed to Simple for mutation_group and sex and the respective Reference Category was ticked. Estimates of effect size and Homogeneity tests were ticked in the Options button dialog and a Significance level of ,05 was accepted. This led to the follow output:

GLM **HSPB1 HSPB5 HSPB5 HSPD1 HSPA1 HSPA2 Ubiquitin SPSS LC3B2**
BY **mutation_group sex** WITH **age_at_operation**
/CONTRAST(**mutation_group**=Simple(1)
/CONTRAST (**sex**)=Simple(1)
/Method=SSTYPE(3)

/INTERCEPT=INCLUDE
/PRINT=ETASQ HOMOGENEITY
/CRITERIA=ALPHA(.05)
/DESIGN=**mutation_group sex age_at_operation.**

Additional Figures

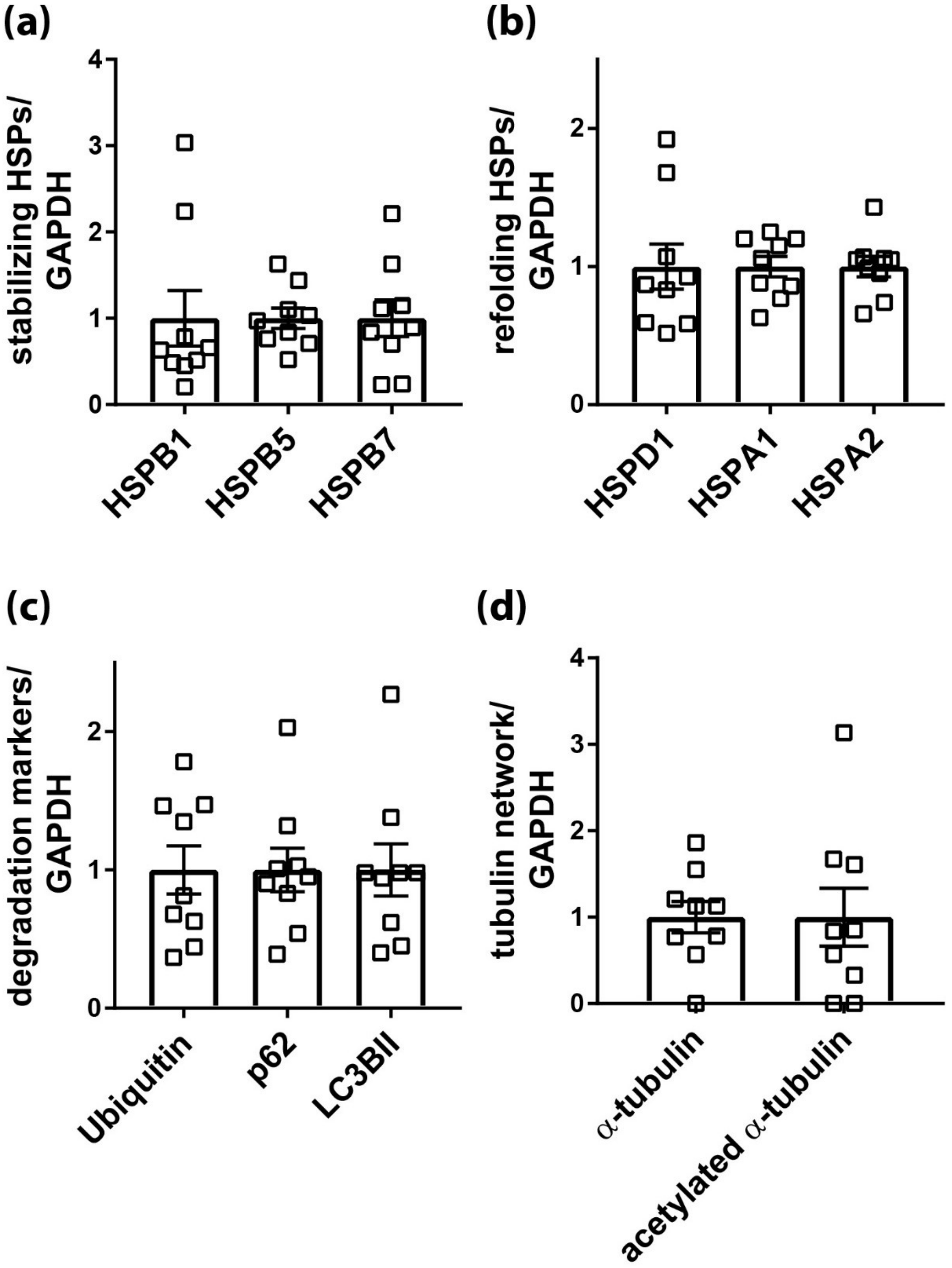

Figure S1. Controls. Distribution of the controls (n=9) for (a) stabilizing HSPs (HSPB1, HSPB5, HSPB7), (b) refolding HSPs (HSPD1, HSPA1, HSPA2), (c) degradation markers (ubiquitin, p62, LC3BII) and (d) tubulin network (α-tubulin and acetylated α-tubulin). The mean of the nine controls was set to one. Each dot in the scatter plots represents an individual sample.

(a)

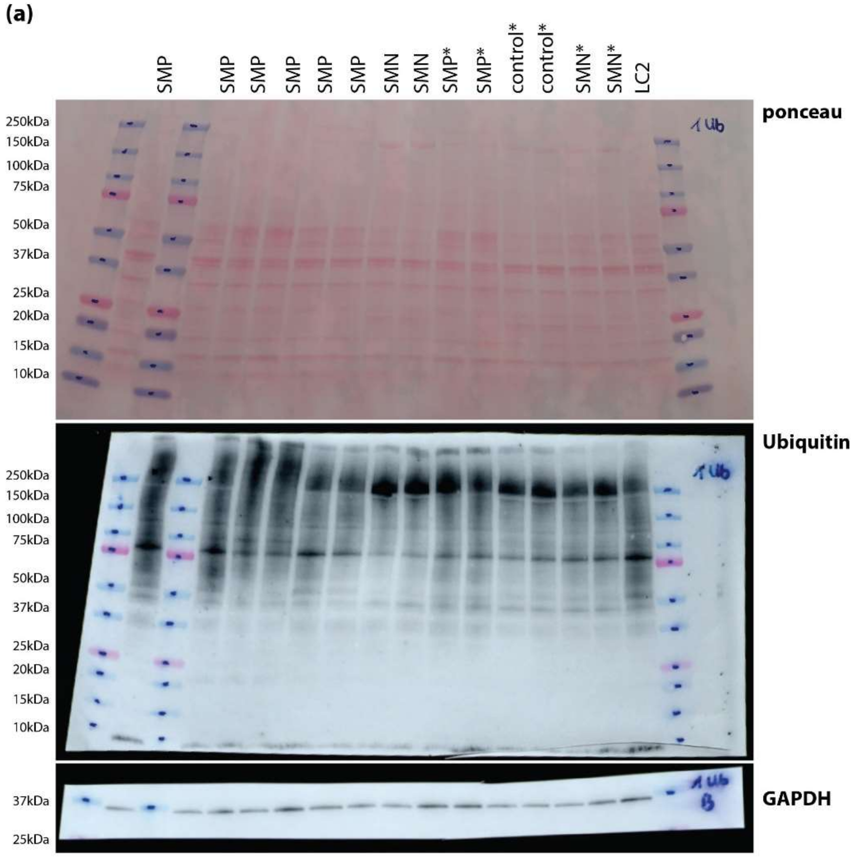
SMP
SMP
SMP
SMP
SMP
SMP
SMN
SMN
SMP*
SMP*
control*
control*
SMN*
SMN*
LC2
ponceau
250kDa
150kDa
100kDa
75kDa
50kDa
37kDa
25kDa
20kDa
15kDa
10kDa
Ubiquitin
GAPDH

(b)

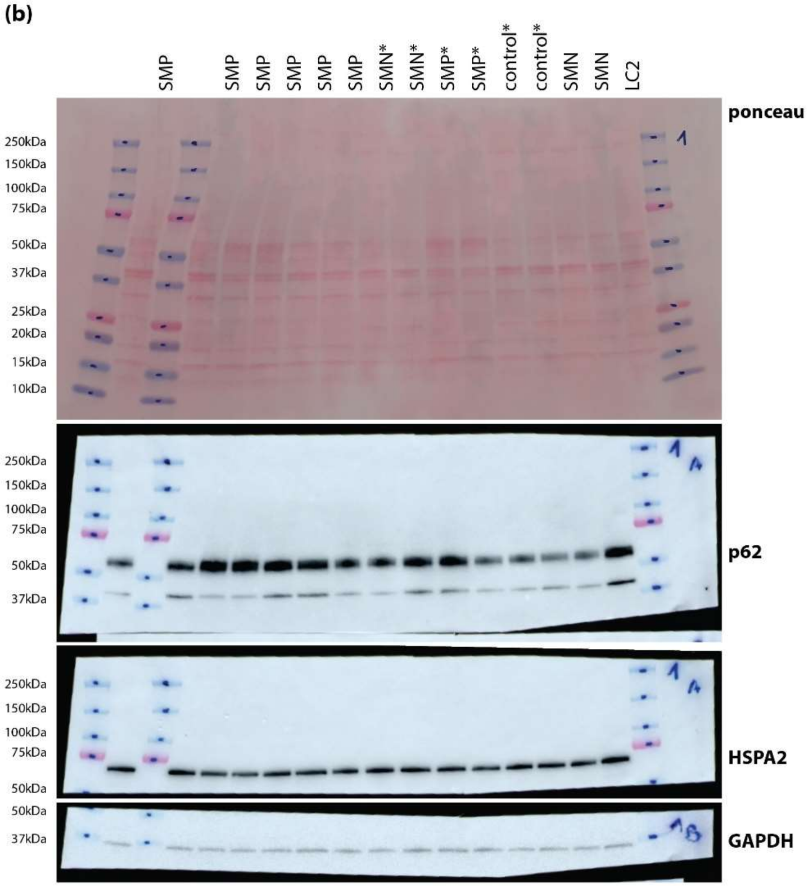
SMP
SMP
SMP
SMP
SMP
SMP
SMN*
SMN*
SMP*
SMP*
control*
control*
SMN
SMN
LC2
ponceau
250kDa
150kDa
100kDa
75kDa
50kDa
37kDa
25kDa
20kDa
15kDa
10kDa
p62
HSPA2
GAPDH

(e)

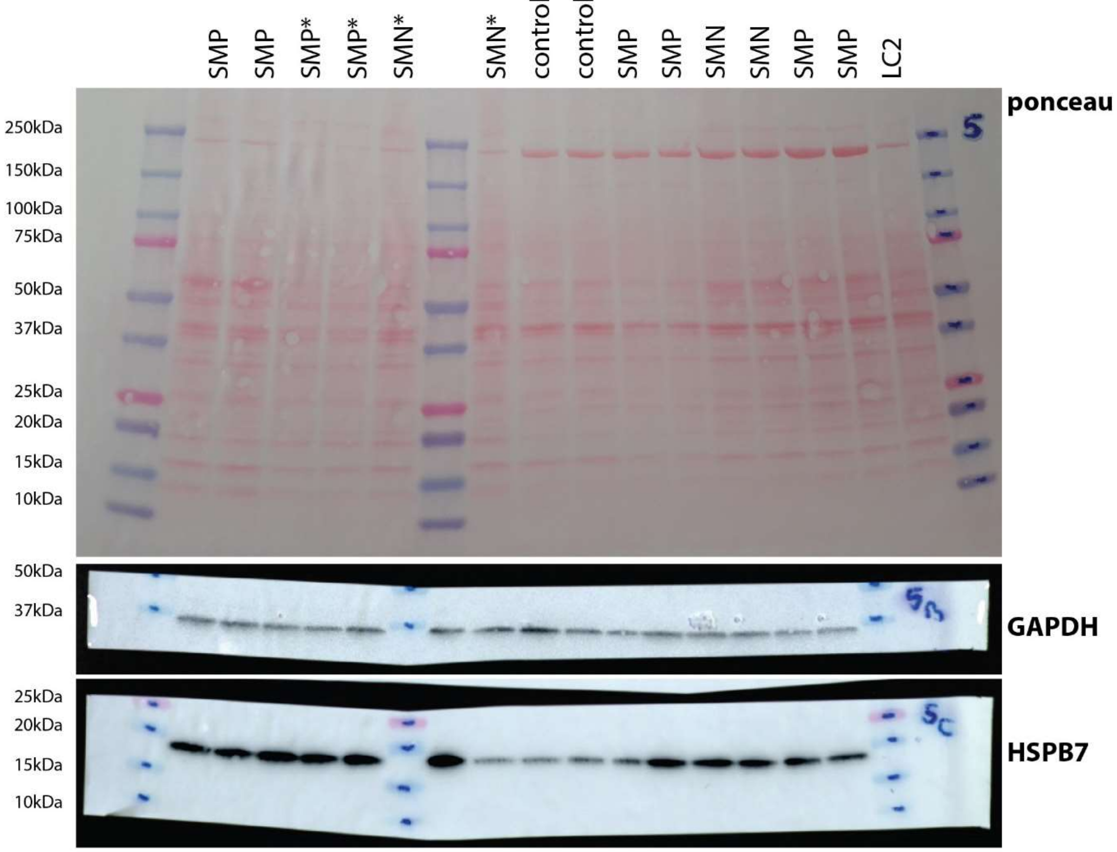
SMP
SMP
SMP*
SMP*
SMN*
SMN*
control*
control*
SMP
SMP
SMN
SMN
SMP
SMP
LC2
ponceau
250kDa
150kDa
100kDa
75kDa
50kDa
37kDa
25kDa
20kDa
15kDa
10kDa
50kDa
37kDa
GAPDH
25kDa
20kDa
15kDa
10kDa
HSPB7

(f)

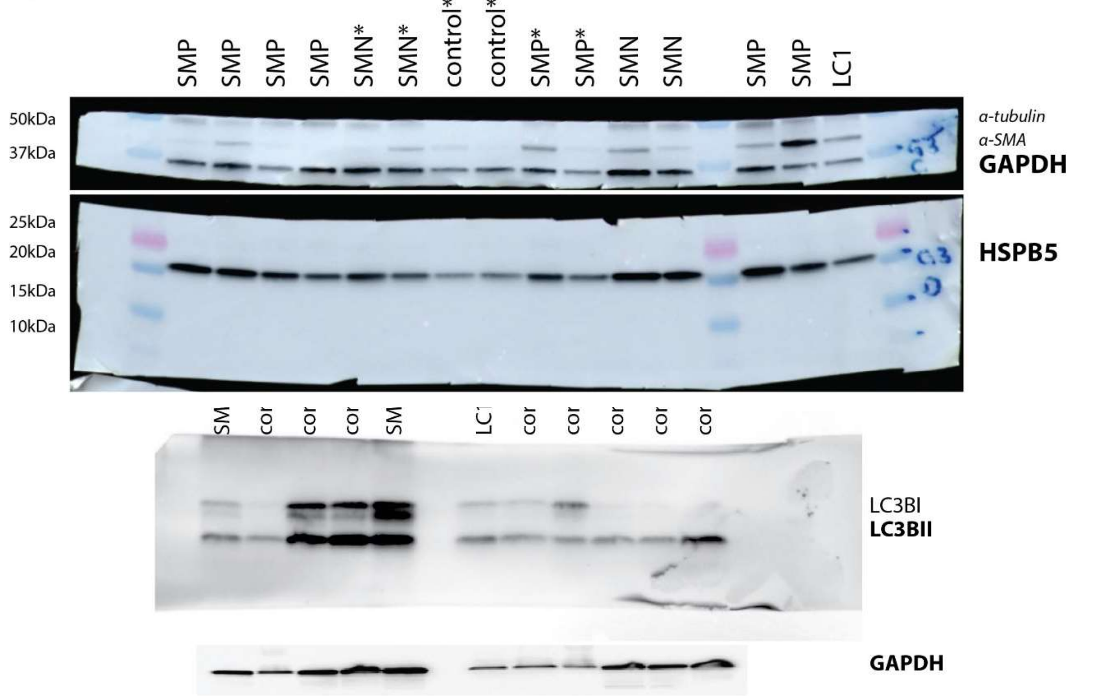
SMP
SMP
SMP
SMP
SMN*
SMN*
control*
control*
SMP*
SMP*
SMN
SMN
SMP
SMP
LC1
50kDa
37kDa
α-tubulin
α-SMA
GAPDH
25kDa
20kDa
15kDa
10kDa
HSPB5
SM
cor
cor
cor
SM
LC
cor
cor
cor
cor
cor
LC3BI
LC3BII
GAPDH

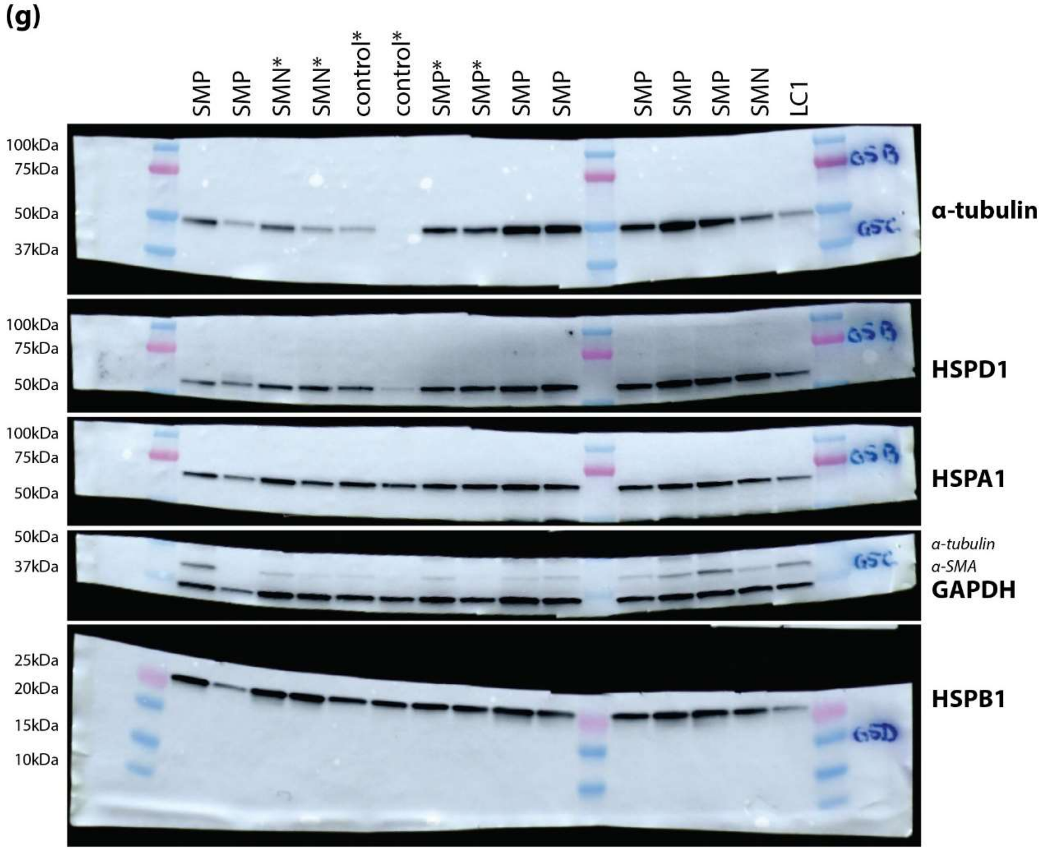

Figure S2. Uncropped full-width pictures of Western blotting membranes. Membranes were often cut to enable blotting for multiple antibodies. (**a**) Full membrane was stained for Ubiquitin and membrane piece (25 kDa – 50 kDa) was thereafter stained for GAPDH. (**b**) Cut membrane piece (> 25 kDa) was stained for p62. Thereafter membrane was cut at 50 kDa and upper part was stained for HSPA2 (> 50 kDa) and lower part (< 50 kDa) for GAPDH. (**c**) Cut membrane piece (> 25 kDa) was stained for acetylated α-tubulin. Thereafter membrane was cut at 50 kDa, lower part (< 50 kDa) was stained for GAPDH. (**d**) Membrane pieces stained for LC3BII and GAPDH. (**e**) Membrane was cut in 3 pieces, middle part (25 kDa – 50 kDa) was stained for GAPDH and lower part (< 25 kDa) for HSPB7. (**f**) Membrane was cut in 3 pieces, middle part (25 kDa – 50 kDa) was stained for GAPDH showing still some remaining signals for the antibodies used before: α-tubulin and α-SMA. The lower part (< 25 kDa) was stained for HSPB5. (**g**) Membrane was cut in 3 pieces, middle part (25 kDa – 120 kDa) was stained for α-tubulin. Thereafter, this piece was cut at 50 kDa. The upper part (50 kDa -120 kDa) was first stained for HSPD1 and then for HSPA1. The lower part (25 kDa – 50 kDa) was stained for GAPDH showing still some remaining signals for the antibodies used before: α-tubulin and α-SMA. The lowest part (< 25 kDa) was stained for HSPB1. Two different loading controls (LC1 and LC2) were used to normalize for differences between various membranes. Asterisk marks the section of samples displayed in the manuscript.

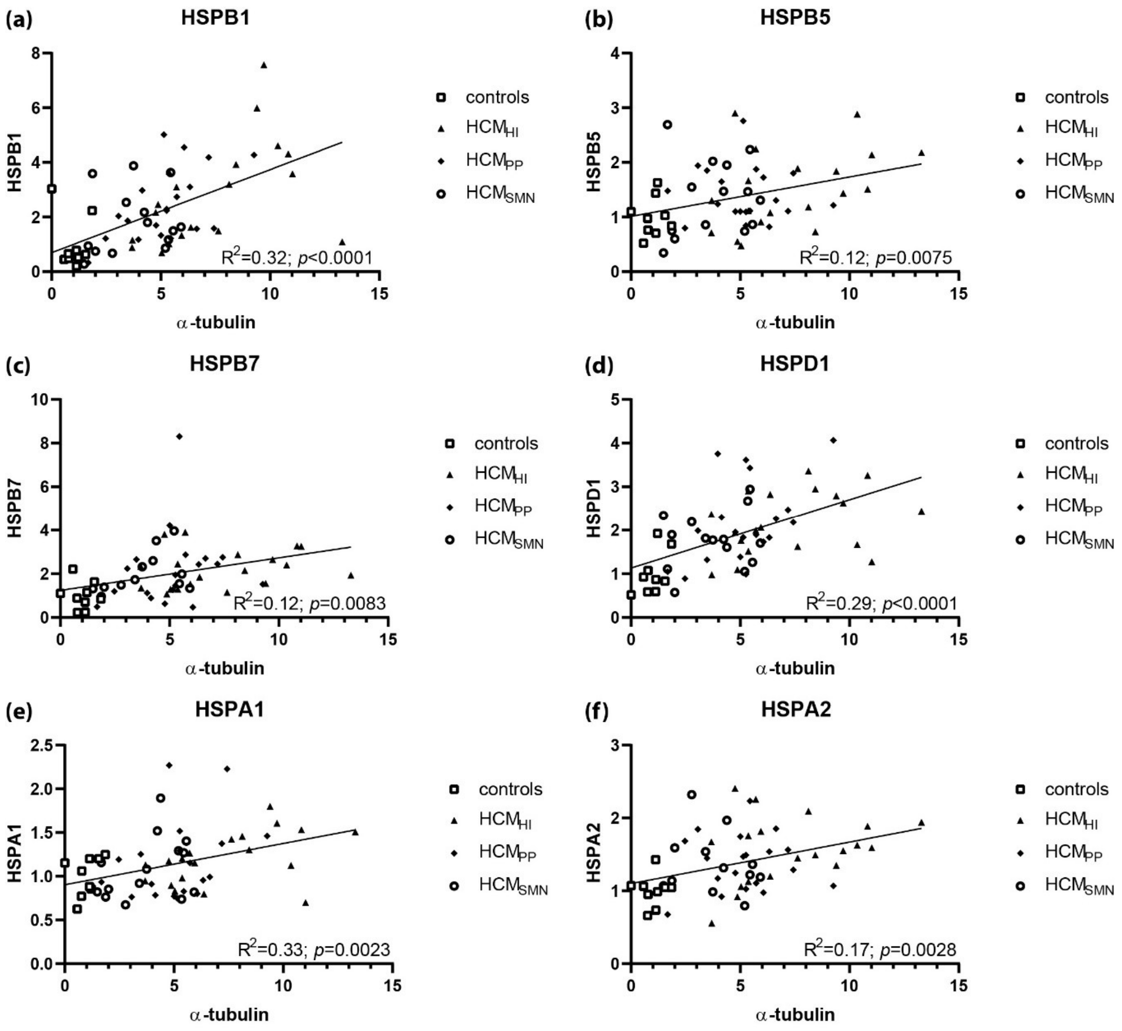

Figure S3. Correlation of HSPs levels and α-tubulin. The levels of (**a**) HSPB1, (**b**) HSPB5, (**c**) HSPB7, (**d**) HSPD1, (**e**) HSPA1 and (**f**) HSPA2 correlated well with α-tubulin. Controls = open squares, HCM$_{SMP}$ = filled circles, HCM$_{SMN}$ = open circles, HCM$_{HI}$ = filled triangles, HCM$_{PP}$ = filled rhomboids. Each dot in the correlation analyses represents an individual sample.

© 2019 by the authors. Submitted for possible open access publication under the terms and conditions of the Creative Commons Attribution (CC BY) license (http://creativecommons.org/licenses/by/4.0/).
